# A viral uveitis masquerade: PCR-confirmed *Listeria monocytogenes* necrotizing chorioretinitis complicated by secondary macular hole

**DOI:** 10.1186/s12348-026-00613-x

**Published:** 2026-06-11

**Authors:** Hai Xuan Ho, Thanh Ngoc Tran, Thoa Thi Doan, Loan Thi Hoang, Lan Huong Thi Tran

**Affiliations:** 1 Vietnam National Eye Hospital, Hanoi, Vietnam; 2 Cung Hong Son International Eye Hospital, Hanoi, Vietnam; 3 Department of Ophthalmology and Optometry, Hanoi Medical University, Hanoi, Vietnam

**Keywords:** *Listeria monocytogenes*, Necrotizing chorioretinitis, Viral uveitis masquerade, Hypertensive uveitis, Macular hole, Polymerase chain reaction, Moxifloxacin

## Abstract

**Background:**

*Listeria monocytogenes* is a rare ocular pathogen, and posterior segment infection is particularly uncommon. When it involves the retina, its presentation may overlap with viral hypertensive uveitis or necrotizing viral retinitis, especially in eyes with unilateral ocular hypertension, iris atrophy, and chronic recurrent inflammation. This diagnostic overlap may delay recognition of an unusual bacterial cause.

**Findings:**

A 23-year-old male presented with progressive visual loss in the left eye after a five-year history of recurrent unilateral ocular redness and previously documented marked ocular hypertension. Examination revealed panuveitis with patchy iris stromal atrophy, pigment dispersion, vitreous inflammation, old peripapillary chorioretinal scars, and an active necrotizing chorioretinal lesion involving the macula, associated with retinal hemorrhage and a secondary macular hole. The initial impression was viral hypertensive uveitis with necrotizing chorioretinitis. Aqueous PCR for herpes simplex virus, varicella-zoster virus, and cytomegalovirus was negative, while bacterial multi-nested PCR detected *L. monocytogenes*. After oral and topical moxifloxacin were added to anti-inflammatory treatment, active intraocular inflammation had clinically resolved by 30 days, the necrotizing chorioretinal lesion evolved into an inactive scar, and the secondary macular hole progressively decreased in size before closing spontaneously. No systemic illness or immunocompromising condition was identified.

**Conclusions:**

This case expands the spectrum of ocular listeriosis in the posterior segment and highlights *L. monocytogenes* as a rare cause of necrotizing chorioretinitis that may masquerade as viral hypertensive uveitis. Aqueous PCR can be decisive when the presentation is atypical or when the response to initial empirical treatment is incomplete. To the best of our knowledge, this is the first reported case of PCR-confirmed *L. monocytogenes* necrotizing chorioretinitis complicated by secondary macular hole formation.

**Supplementary Information:**

The online version contains supplementary material available at 10.1186/s12348-026-00613-x.

## Findings

### Background

*Listeria monocytogenes* is a facultative intracellular, gram-positive bacterium responsible for listeriosis, which occurs more frequently in immunocompromised individuals but may also affect otherwise healthy patients. Although best known as a food-borne pathogen capable of causing invasive systemic disease, ocular involvement remains rare [1]. Reported manifestations range from conjunctivitis, keratitis, and anterior uveitis to vision-threatening endogenous endophthalmitis [2]. Posterior segment involvement is much less frequently reported. In particular, necrotizing chorioretinitis caused by *L. monocytogenes* appears to be exceptionally rare and may clinically mimic more common entities, such as viral uveitis or necrotizing viral retinitis. This diagnostic overlap is especially challenging when anterior segment findings, including unilateral ocular hypertension, iris atrophy, and pigment dispersion, suggest a herpetic phenotype. We report a PCR-confirmed case of *L. monocytogenes* necrotizing chorioretinitis in a healthy young male who initially presented as a viral uveitis masquerade. The disease followed an indolent course over five years before diagnosis and ultimately resulted in secondary macular hole formation after macular chorioretinal necrosis.

### Case report

A 23-year-old previously healthy male restaurant chef from a rural province presented to the Vietnam National Eye Hospital with progressive blurred vision in his left eye for two weeks. During this current episode, he reported no ocular pain or redness. He had no history of ocular trauma or prior ocular surgery.

His ocular history was notable for recurrent episodes of unilateral redness in the left eye beginning five years earlier, initially without pain and treated intermittently at a local clinic with unidentified topical medications, which he later self-administered during recurrent episodes with transient relief. After relocating for work, he was evaluated in the Glaucoma Service at a tertiary referral center, where markedly asymmetric intraocular pressure was documented, measuring 52 mmHg in the left eye and 20 mmHg in the right eye, with best-corrected visual acuity of 6/6 in both eyes. According to the available records, a diagnosis of primary open-angle glaucoma in the left eye was made at that time, although secondary glaucoma related to prior intraocular inflammation or corticosteroid-containing topical medications could not be excluded retrospectively. He was treated with topical brinzolamide/timolol (Azarga), achieving good intraocular pressure control. He remained under glaucoma follow-up for several years; however, the available historical records did not contain detailed posterior segment documentation. He was advised to discontinue treatment two years before the current presentation because intraocular pressure remained controlled off medication, after which he was lost to follow-up.

He denied systemic symptoms or known systemic disease. There was no recalled history of varicella or herpes zoster infection. He denied animal exposure and reported no habitual intake of undercooked food, although he noted frequent consumption of processed meats, including canned pork and sausages.

**Fig. 1:**
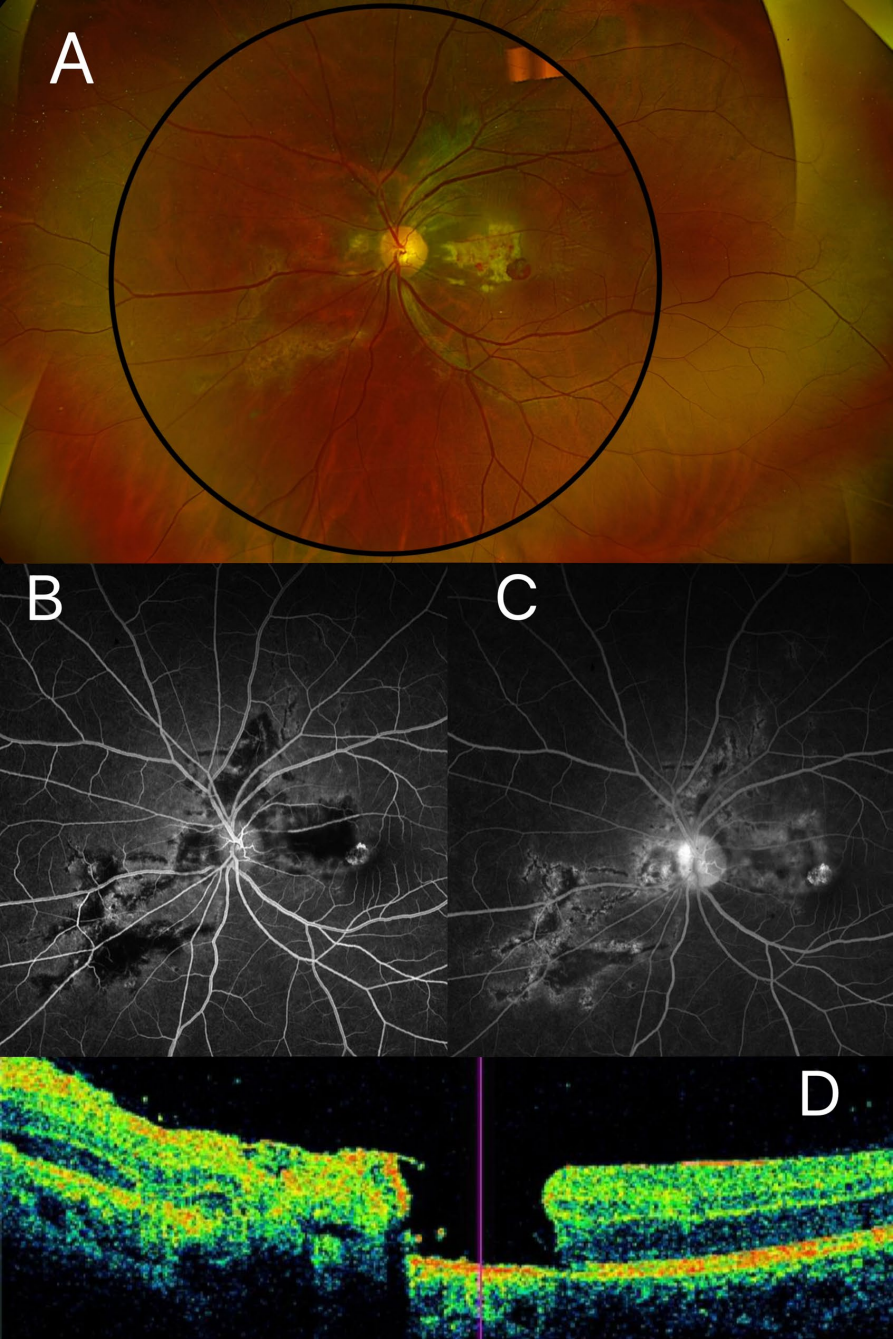
Baseline posterior segment imaging of the left eye. **A**. Ultra-widefield color fundus photograph shows a creamy yellow-white necrotizing chorioretinal lesion involving the macular region, associated with flame-shaped retinal hemorrhage and multiple old peripapillary serpiginous-like chorioretinal scars. The circled area highlights the posterior pole, including the active lesion and peripapillary scars. **B**, **C**. Fluorescein angiography shows representative earlier and later angiographic frames, with irregular hyperfluorescence, leakage, and staining in the macular region, corresponding to the active necrotizing lesion. A focal foveal hyperfluorescent defect is consistent with the full-thickness macular hole. The old peripapillary scars show mottled staining without definite active leakage. **D**. Macular optical coherence tomography confirms a full-thickness macular hole with an associated epiretinal membrane. The nasal margin shows retinal thinning with hyperreflectivity, architectural disorganization, and loss of normal lamination, consistent with necrotizing chorioretinal damage

On examination, BCVA was 6/6 in the right eye and counting fingers at 0.3 m in the left eye. Intraocular pressure was 16 mmHg in the right eye and 15 mmHg in the left eye. No relative afferent pupillary defect was detected. Anterior segment examination of the left eye showed mild ciliary injection, mild corneal edema, scattered small-to-medium keratic precipitates with pigment deposits, and anterior chamber inflammation with 3+ cells and 2+ flare, accompanied by dispersed pigment debris without posterior synechiae. The iris showed patchy stromal atrophy, predominantly around the pupillary margin. The pupil remained round and reactive to light. Lens examination disclosed inferior glaukomflecken and mild scattered cortical cataractous changes. Anterior vitreous inflammation was present with 3+ anterior vitreous cells, 2+ vitreous haze, and several inferior snowball opacities. Fundus examination revealed a creamy yellow-white necrotizing chorioretinal lesion with indistinct edematous borders involving the macular region, associated with flame-shaped retinal hemorrhages and a concurrent macular hole. Multiple old serpiginous-like chorioretinal atrophic scars were also observed adjacent to the optic disc, extending into the superior and inferonasal retina (Fig. 1A). The right eye was unremarkable. Extraocular motility and adnexal examination were normal. Systemic evaluation by an internist was unremarkable.

Ultra-widefield fluorescein angiography demonstrated irregular hyperfluorescence with leakage and staining in the macular region, corresponding to the active necrotizing chorioretinal lesion (Fig. 1B, C). A focal foveal hyperfluorescent defect corresponded to the full-thickness macular hole, while adjacent areas of hypofluorescence were consistent with blocked fluorescence from hemorrhage and pigmentary change. The old peripapillary serpiginous-like lesions showed mottled staining without definite active leakage, suggesting inactive chorioretinal scarring. Spectral-domain optical coherence tomography confirmed a full-thickness macular hole with an associated epiretinal membrane (Fig. 1D). The nasal margin of the hole showed retinal thinning, architectural disorganization, and loss of normal lamination, consistent with necrotizing chorioretinal damage. In contrast, the temporal retina appeared relatively thickened and distorted, likely reflecting epiretinal membrane traction and associated retinal edema.

The initial diagnosis was left-eye panuveitis with necrotizing chorioretinitis and secondary macular hole, with viral etiology initially considered most likely. Given the necrotizing posterior segment inflammation, anterior chamber fluid was urgently obtained for PCR testing for herpes simplex virus, varicella-zoster virus, and cytomegalovirus. Because of the atypical and indolent presentation, an infectious cause other than viral etiology was also considered, and the aqueous sample was simultaneously sent for xGen™ multi-nested PCR bacterial screening. A routine uveitis work-up, including testing for syphilis and tuberculosis, was performed. While awaiting the results, treatment was started with oral acyclovir 800 mg five times daily, oral prednisolone at an equivalent dose of 0.5 mg/kg/day, prednisolone acetate 1% eye drops six times daily in the left eye, and atropine 1% twice daily. The patient requested outpatient management and was therefore reviewed daily.

After three days of treatment, symptoms and ocular findings remained largely unchanged. Hospital admission was recommended and accepted. Because a bacterial etiology remained a concern, empirical oral moxifloxacin 400 mg once daily and topical moxifloxacin 0.5% six times daily were added. The following day, the patient reported subjective improvement, and slit-lamp examination showed reduced ciliary injection and a decrease in anterior chamber cells from 3+ to 2+. However, the anterior chamber flare and fundus findings were unchanged.

Five days after initiation of moxifloxacin, the left eye showed clear clinical improvement. BCVA in the left eye improved to counting fingers at 1.5 m. Ciliary injection and corneal edema had resolved. Keratic precipitates and pigment deposits were reduced, anterior chamber cells decreased to 1+, while flare persisted at 2+. Vitreous inflammation also improved, with 2+ vitreous cells and 1+ haze, and the inferior snowball opacities became smaller. The necrotizing chorioretinal lesion appeared less active, with reduced retinal edema, a more clearly demarcated border, and early scarring. The secondary macular hole and old peripapillary chorioretinal scars remained stable.

The laboratory results subsequently returned negative for HSV, VZV, CMV, tuberculosis, syphilis, HIV, and hepatitis B. Complete blood count, liver function, and renal function tests were within normal limits. PCR of the aqueous fluid was positive for *Listeria monocytogenes*. After the microbiological diagnosis was established, treatment options for ocular listeriosis were discussed with the patient, including systemic intravenous antibiotic therapy and intravitreal antimicrobial treatment. As the eye had already shown early clinical improvement after moxifloxacin was introduced, and because the patient declined invasive therapy after counseling, treatment was continued with oral moxifloxacin 400 mg once daily, topical moxifloxacin 0.5% six times daily, atropine 1% twice daily, and oral and topical corticosteroids. Oral acyclovir was discontinued.

**Fig. 2:**
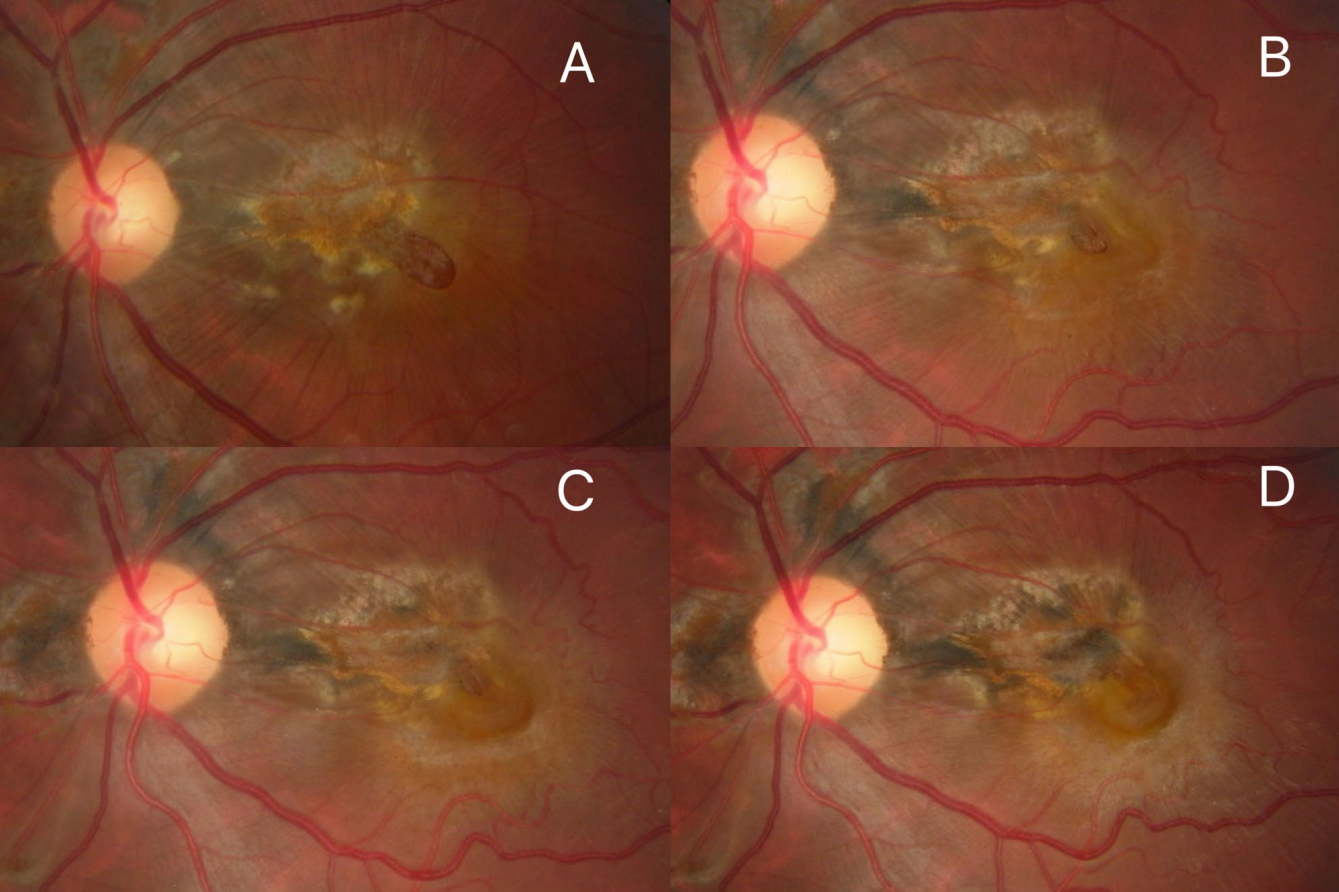
Color fundus changes during follow-up. **A** Twelve days after initiation of moxifloxacin, the necrotizing lesion appears less active, with reduced edema and more clearly demarcated borders. **B** Twenty days after treatment, the lesion shows further drying, early pigmentary change, and an apparent reduction in the foveal defect. **C** Thirty days after treatment, the lesion has evolved into an inactive white scar with increasing pigmentary change, while the foveal defect appears smaller. **D** At the final follow-up, the scar remains dry and sharply demarcated, without recurrent inflammatory activity

**Fig. 3:**
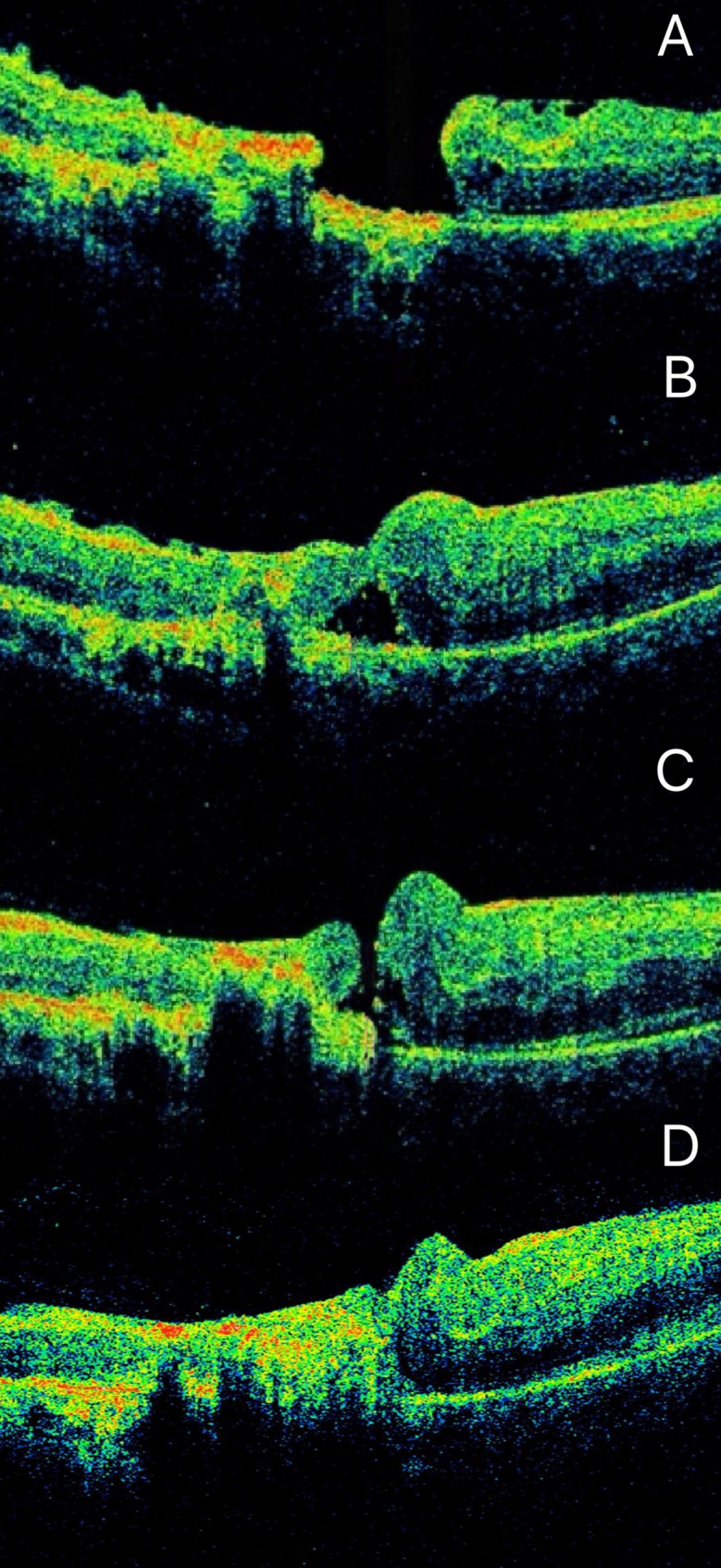
Serial optical coherence tomography changes during follow-up. **A** Twelve days after initiation of moxifloxacin, optical coherence tomography shows a persistent full-thickness macular hole. The previously active nasal retinal lesion appears inactive, with retinal thinning and atrophic change, without obvious residual signs of active retinal inflammation. **B** Twenty days after treatment, epiretinal membrane traction is associated with elevation and thickening of the temporal edge of the hole. The macular hole appears smaller, with early bridging across the defect, while inflammatory changes in the nasal retina have regressed. **C** Thirty days after treatment, the hole further decreases in size. Increased hyperreflectivity and elevation at the outer retinal layers suggest ongoing scar-related change. Early tissue migration toward the defect is also noted, consistent with progressive spontaneous closure. **D** At the final follow-up, the macular hole is completely closed. The foveal region is replaced by a densely hyperreflective scar-like tissue complex, consistent with cicatricial closure. Temporal retinal thickening persists, likely related to residual epiretinal membrane traction

Twelve days after starting moxifloxacin, BCVA in the left eye improved to counting fingers at 4 m, with normal intraocular pressure. The right eye remained unchanged. There was no ciliary injection, the cornea was clear, and only a few old keratic precipitates with pigment deposits remained (Additional file 1: Fig. S1). Anterior chamber inflammation decreased to 0.5+ cells with 1+ flare. Vitreous inflammation continued to subside, with 1+ cells and 1+ haze, and the snowball opacities further regressed. The necrotizing chorioretinal lesion appeared clinically inactive, with a dry, yellow-white, sharply demarcated scar (Fig. 2A). The secondary macular hole persisted, with foveal distortion related to traction from a secondary epiretinal membrane (Fig. 3A), while the old peripapillary scars remained stable. As the patient felt better, he requested discharge and preferred to continue treatment as an outpatient. He was discharged with close follow-up and continued oral moxifloxacin 400 mg once daily, tapering oral prednisolone and topical prednisolone acetate, topical moxifloxacin 0.5% four times daily, and atropine 1% twice daily.

Twenty days after starting moxifloxacin, BCVA in the left eye further improved to 6/120, with normal intraocular pressure, and the right eye remained stable. The anterior segment was quiet, with no ciliary injection, a clear cornea, and only residual old keratic precipitates with pigment deposits. Anterior chamber cells were no longer observed, although 1+ flare persisted. Vitreous inflammation improved further, with 0.5+ cells and 1+ haze, and the inferior snowball opacities became less prominent. The necrotizing chorioretinal scar was dry and sharply demarcated, with early pigmentary change (Fig. 2B). The secondary macular hole had decreased in size, with partial tissue bridging, suggesting early spontaneous closure (Fig. 3B). The foveal contour remained distorted by epiretinal membrane traction, and the old peripapillary scars were unchanged. The patient continued outpatient treatment with oral moxifloxacin 400 mg once daily, tapering oral prednisolone and topical prednisolone acetate, and tapering topical moxifloxacin 0.5%. Atropine was discontinued.

Thirty days after starting moxifloxacin, BCVA in the left eye remained at 6/120, with normal intraocular pressure. The right eye remained unchanged. The left eye was clinically quiet, with no ciliary injection, a clear cornea, and only residual pigment deposits on the posterior corneal surface. There were no anterior chamber cells, with only faint residual flare. Vitreous inflammation had resolved, with no vitreous cells or haze, and no new snowball opacities were observed. The necrotizing chorioretinal lesion remained inactive and continued to mature into a dry, white, sharply demarcated scar with increasing pigmentary change (Fig. 2C). The macular hole continued to decrease in size, with progressive spontaneous closure, although foveal distortion from the epiretinal membrane persisted (Fig. 3C). The patient completed the corticosteroid taper. Oral moxifloxacin was discontinued one week after steroid cessation, and all topical medications were stopped. Because he lived far from our hospital, he was instructed to monitor the left eye closely and seek urgent ophthalmic care if redness, pain, or visual deterioration recurred.

One month after discontinuing all medications, he returned for follow-up. BCVA in the left eye had improved to 6/48, with normal intraocular pressure. The eye remained quiet, with no ciliary injection, a clear cornea, and only residual pigment deposits on the posterior corneal surface. There were no anterior chamber cells or clinically significant flare. Vitreous inflammation remained resolved, with no vitreous cells, no haze, and no recurrent snowball opacities. The necrotizing chorioretinal lesion was fully inactive, leaving a dry, sharply demarcated white scar with pigmentary change (Fig. 2D). The secondary macular hole had closed spontaneously, although the foveal contour remained distorted by the epiretinal membrane (Fig. 3D). The old peripapillary scars and the right eye remained stable. Given the stable course and the patient’s distance from our hospital, he was advised to maintain good food hygiene and avoid undercooked foods and ready-to-eat processed meats. He was also advised to continue self-monitoring and attend regular ophthalmic follow-up locally. At the time of manuscript preparation, he reported that the eye remained stable without recurrence.

## Discussion

*Listeria monocytogenes* is a facultative intracellular, gram-positive bacterium known as a foodborne pathogen, but its clinical relevance extends beyond gastrointestinal disease. It can invade epithelial cells, survive intracellularly, and spread hematogenously, including within infected phagocytes [3–5]. Its ability to cross epithelial and endothelial barriers, especially in CNS invasion, explains its neuroinvasive nature [2, 6]. This is relevant to ocular pathogenesis because the retina is an anatomical and developmental extension of the central nervous system, making posterior segment infection biologically plausible, though rarely.

Ocular listeriosis is uncommon, and its recognition may be challenging when the initial presentation does not resemble a typical invasive bacterial infection. The present case is unusual not only because the patient was a young immunocompetent adult, but also because the disease behaved as a long-standing viral masquerade. His previous episodes of unilateral ocular redness and marked ocular hypertension had been managed as glaucoma according to the available records. However, the later findings of patchy iris stromal atrophy, pigment dispersion, old peripapillary chorioretinal scars, and PCR-proven *L. monocytogenes* raise the possibility that at least part of this earlier course represented smoldering infectious hypertensive uveitis rather than primary glaucoma alone. The absence of detailed posterior segment documentation in the historical records, together with preserved visual acuity at that time, may have contributed to delayed recognition, particularly if earlier lesions spared the fovea. In contrast, the current episode involved the posterior pole, including the macula, leading to visual decline, recognition of necrotizing chorioretinitis, and subsequent microbiological confirmation. Although no gastrointestinal illness was reported, frequent consumption of processed meats represented a plausible exposure; however, no direct food source was identified.

The detection of *L. monocytogenes* was unexpected. Most reported ocular cases have involved severe anterior segment disease, hypertensive uveitis, pigmented hypopyon, or endogenous endophthalmitis, whereas predominant posterior segment involvement remains much less frequently described [2, 7, 8]. The closest posterior segment report we identified was described by Huismans et al. in 1986: a 16-year-old girl developed acute chorioretinitis after a flu-like illness with severe headache, with only mild anterior chamber and vitreous inflammation. The active lesion was located near the superior temporal arcade, approximately two disc diameters temporal to the optic disc, and was associated with a neuroretinitis-like macular star. She was initially treated empirically with doxycycline and systemic corticosteroids, but the diagnosis was later established by Listeria-Widal agglutination, after which treatment was redirected toward *Listeria*-targeted antimicrobial therapy with a favorable visual outcome [8]. Our case differs in several important ways. The patient had no systemic symptoms, was an immunocompetent young adult, and had a five-year indolent course that initially resembled viral hypertensive uveitis. The diagnosis was confirmed by aqueous PCR rather than serology. Moreover, the active necrotizing lesion involved the macula, resulting not only in chorioretinal scarring but also in secondary macular hole formation. To the best of our knowledge, macular hole formation has not previously been reported as a structural complication of *L. monocytogenes* necrotizing chorioretinitis. This case, therefore, expands the recognized posterior segment spectrum of ocular listeriosis and supports considering *L. monocytogenes* in atypical necrotizing chorioretinitis, particularly when the clinical course is indolent, the lesion distribution is unusual, or inflammation fails to improve adequately after initial empirical treatment, whether directed against viral or bacterial causes.

There is no standardized regimen for ocular *Listeria monocytogenes* infection, especially when the disease presents as necrotizing chorioretinitis rather than endophthalmitis. Treatment is generally extrapolated from invasive listeriosis and central nervous system infection, for which high-dose ampicillin or amoxicillin, often combined with gentamicin, remains the conventional first-line approach [4, 5]. In reported cases of *Listeria* endophthalmitis, management has often required aggressive local therapy, including intravitreal antibiotics such as vancomycin, gentamicin, or amikacin, and, in selected cases, vitrectomy or anterior chamber washout [1, 2, 9].

However, antimicrobial selection in ocular listeriosis is complicated by two barriers: the intracellular biology of *L. monocytogenes* and the blood-retinal barrier [1, 2, 6]. Moxifloxacin was a biologically plausible option in this case because experimental studies have shown rapid bactericidal activity against intracellular *L. monocytogenes* [2, 3], with activity comparable to ampicillin plus gentamicin in experimental *Listeria* meningitis [10]. Human pharmacokinetic studies have shown that orally administered moxifloxacin achieves measurable intraocular concentrations even in noninflamed eyes, with repeated once-daily dosing producing higher intravitreal levels than a single oral dose [11]. Although fluoroquinolones are not established as standard treatment for invasive listeriosis, successful clinical use has been reported in selected systemic cases [5, 12]. These reports, together with experimental data, provide a rationale for considering fluoroquinolones, particularly moxifloxacin, in selected cases where tissue penetration and intracellular bactericidal activity are desirable.

Because the patient improved clinically and declined intravenous or intravitreal therapy after counseling, oral and topical moxifloxacin were continued with corticosteroids under close follow-up; no adverse effects attributable to moxifloxacin were observed. The duration of therapy was individualized. Because no established treatment duration exists for ocular *Listeria* necrotizing chorioretinitis, we extrapolated from invasive listeriosis and central nervous system infection, in which antibiotic courses commonly range from approximately 2 to 6 weeks and may be extended in severe disease [1, 6]. In this patient, moxifloxacin was continued for about five weeks in total, including one additional week after corticosteroid cessation, to ensure sustained quiescence of the chorioretinal lesion while anti-inflammatory therapy was withdrawn. To our knowledge, this is the first reported case of PCR-confirmed *L. monocytogenes* necrotizing chorioretinitis and secondary macular hole formation showing a favorable clinical course after a moxifloxacin-containing regimen. This observation suggests that moxifloxacin may be a useful alternative or adjunctive option in selected ocular listeriosis, particularly when posterior segment penetration and intracellular activity are desirable.

This report has several limitations. The diagnosis was based on aqueous PCR and clinical correlation, without culture confirmation, antimicrobial susceptibility testing, or histopathology. The earlier five-year course was reconstructed retrospectively from available clinical records, which did not include detailed posterior segment documentation. In addition, the favorable outcome cannot be attributed to moxifloxacin alone, as treatment included concomitant corticosteroids and no controlled comparison was possible. These limitations are inherent to a single rare case and should be considered when interpreting the diagnostic and therapeutic implications. Therefore, although the present case suggests that moxifloxacin may be a useful alternative or adjunctive option in selected cases of ocular listeriosis, it cannot establish moxifloxacin as standard therapy, and further clinical evidence is required.

## Supplementary Information

Below is the link to the electronic supplementary material.

Supplementary Material 1: Day-12 anterior segment photograph of the left eye. Slit-lamp photograph obtained 12 days after initiation of moxifloxacin shows resolved anterior segment inflammation, with no ciliary injection and a clear cornea. Residual old keratic precipitates with pigment deposits and patchy iris stromal atrophy around the pupillary margin are visible

## Data Availability

No datasets were generated or analysed during the current study.

## Abbreviations

BCVA: Best-corrected visual acuity
CMV: Cytomegalovirus
CNS: Central nervous system
FA: Fluorescein angiography
HIV: Human immunodeficiency virus
HSV: Herpes simplex virus
IOP: Intraocular pressure
OCT: Optical coherence tomography
PCR: Polymerase chain reaction
VZV: Varicella-zoster virus
